# Development of a proteomic signature associated with severe disease for patients with COVID-19 using data from 5 multicenter, randomized, controlled, and prospective studies

**DOI:** 10.1038/s41598-023-46343-1

**Published:** 2023-11-20

**Authors:** Sandra Castro-Pearson, Sarah Samorodnitsky, Kaifeng Yang, Sahar Lotfi-Emran, Nicholas E. Ingraham, Carolyn Bramante, Emma K. Jones, Sarah Greising, Meng Yu, Brian T. Steffen, Julia Svensson, Eric Åhlberg, Björn Österberg, David Wacker, Weihua Guan, Michael Puskarich, Anna Smed-Sörensen, Elizabeth Lusczek, Sandra E. Safo, Christopher J. Tignanelli

**Affiliations:** 1 Division of Biostatistics, School of Public Health, University of Minnesota, Minneapolis, MN USA; 2 Department of Medicine, University of Minnesota, Minneapolis, MN USA; 3 Department of Surgery, University of Minnesota, 420 Delaware St SE, Minneapolis, MN 55455 USA; 4 School of Kinesiology, University of Minnesota, Minneapolis, MN USA; 5 Division of Immunology and Allergy, Department of Medicine Solna, Center for Molecular Medicine, Karolinska Institutet and Karolinska University Hospital, Stockholm, Sweden; 6 Department of Emergency Medicine, University of Minnesota, Minneapolis, MN USA; 7 Department of Emergency Medicine, Hennepin County Medical Center, Minneapolis, MN USA; 8 Institute for Health Informatics, University of Minnesota, Minneapolis, MN USA

**Keywords:** Computational biology and bioinformatics, Medical research, Respiratory distress syndrome

## Abstract

Significant progress has been made in preventing severe COVID-19 disease through the development of vaccines. However, we still lack a validated baseline predictive biologic signature for the development of more severe disease in both outpatients and inpatients infected with SARS-CoV-2. The objective of this study was to develop and externally validate, via 5 international outpatient and inpatient trials and/or prospective cohort studies, a novel baseline proteomic signature, which predicts the development of moderate or severe (vs mild) disease in patients with COVID-19 from a proteomic analysis of 7000 + proteins. The secondary objective was exploratory, to identify (1) individual baseline protein levels and/or (2) protein level changes within the first 2 weeks of acute infection that are associated with the development of moderate/severe (vs mild) disease. For model development, samples collected from 2 randomized controlled trials were used. Plasma was isolated and the SomaLogic SomaScan platform was used to characterize protein levels for 7301 proteins of interest for all studies. We dichotomized 113 patients as having mild or moderate/severe COVID-19 disease. An elastic net approach was used to develop a predictive proteomic signature. For validation, we applied our signature to data from three independent prospective biomarker studies. We found 4110 proteins measured at baseline that significantly differed between patients with mild COVID-19 and those with moderate/severe COVID-19 after adjusting for multiple hypothesis testing. Baseline protein expression was associated with predicted disease severity with an error rate of 4.7% (AUC = 0.964). We also found that five proteins (Afamin, I-309, NKG2A, PRS57, LIPK) and patient age serve as a signature that separates patients with mild COVID-19 and patients with moderate/severe COVID-19 with an error rate of 1.77% (AUC = 0.9804). This panel was validated using data from 3 external studies with AUCs of 0.764 (Harvard University), 0.696 (University of Colorado), and 0.893 (Karolinska Institutet). In this study we developed and externally validated a baseline COVID-19 proteomic signature associated with disease severity for potential use in both outpatients and inpatients with COVID-19.

## Introduction

The 2019 novel coronavirus disease (COVID-19), caused by the severe acute respiratory syndrome coronavirus 2 (SARS-CoV-2), has persisted as one of the leading causes of death worldwide^1,2^. While significant progress has been made in preventing severe disease through the development of effective vaccines, we still lack a validated baseline predictive biologic signature for the development of severe disease in both outpatients and inpatients infected with COVID-19. Previous studies are limited by focusing on patients that required hospitalization or an emergency department (ED) visit and thus skewed towards sicker patients. Additionally, previous studies were limited by low sample sizes. The ALPS-COVID trials were 2 multi-center randomized controlled trials which evaluated the efficacy of losartan as a treatment in patients with COVID-19^3,4^. In addition to populations present in other proteomic signature generation studies, the outpatient ALPS-COVID trial also enrolled symptomatic outpatients that did not require any ED or urgent care visits. Thus, the objective of this study was to develop and externally validate, via 3 additional international outpatient and inpatient trials, a novel baseline proteomic signature which predicted the development of moderate or severe (vs mild) disease in patients with COVID-19 from a proteomic analysis of 7000 + proteins. The secondary objective was exploratory, to identify (1) individual baseline protein levels and/or (2) protein level changes within the first 2 weeks of acute infection that are associated with the development of moderate/severe (vs mild) disease.

## Methods

### Proteomic signature development: patient population

This study was approved by the Advarra central institutional review board (Losartan for COVID-19 Outpatient trial: Advarra Pro00042760; Losartan for COVID-19 Inpatient trial: Advarra Pro00042757) and all participants provided written informed consent. All methods were performed in accordance with relevant guidelines and regulations. All data were fully de-identified prior to analysis. Clinical trial data is available from the corresponding author on reasonable request and also publicly available on Vivli-Registry ID NCT04312009 (https://vivli.org/resources/resources/).

Patients who participated provided blood samples for one of two multi-center, placebo-controlled randomized clinical trials to evaluate the efficacy of losartan in hospitalized and non-hospitalized patients with COVID-19^3,4^. At each day of follow up, COVID-19 disease severity was recorded using a modification of the World Health Organization (WHO) ordinal scale: (0) Death, (1) Hospitalized, on invasive mechanical ventilation or ECMO, (2) Hospitalized on non-invasive mechanical ventilation or high flow devices, (3) Hospitalized requiring Oxygen, (4) Hospitalized, not on oxygen, (5) Not hospitalized. To handle missing day-15 outcomes, an a priori decision was made to assign all outpatients missing a day 15 outcome an outcome of 5. Inpatients who were missing a day 15 outcome had their last observed outcome carried forward.

### Primary outcome

We constructed a binary outcome, “mild” vs. “moderate/severe,” to reflect the worst severity experienced by each individual. Patients were categorized as having “mild” disease if they had a WHO score of 5. “Moderate/severe disease” was defined as at least one WHO score of 4 or lower.

### Proteomics data, quality control, preprocessing, filtering, and normalization

Blood samples were collected at baseline (at the time of study randomization) and at day 15 following randomization. Samples were collected in Ethylenediaminetetraacetic acid (EDTA) (96.5%) or citrated (3.5%) tubes and plasma was extracted as per trial protocol within 6 h of collection. Plasma was frozen at – 80 °C and batch analyzed at the end of the trial. The SomaLogic (Boulder, CO) SomaScan aptamer panel platform which measures 7000 + proteins was used^5^. The SomaLogic SomaScan aptamer panel reports abundances of aptamers which target specific proteins. Multiple aptamers may map to the same protein. All analyses were done on the SomaScan aptamers and results are reported based on the corresponding protein target.

We checked for the presence of a batch effect from the EDTA and citrated storage types using guided principal components analysis (gPCA)^6^. We applied gPCA to sample collection day and tube type. We combined the p-values across the days using Fisher’s method (p = 0.53). As pharmacodynamics studies did not identify a significant difference in renin–angiotensin–aldosterone system (RAAS) signaling between the losartan and placebo groups, we merged data from both outpatients and inpatients who were randomized to either cohort. We removed any proteins corresponding to non-human protein targets, leaving 7301 proteins in the dataset. We removed all quality control samples and 18 individuals for whom we did not have clinical data available. We also removed 8 samples whose median signal normalization scale factor at 20% mix fell below 0.26. This left 138 subjects for downstream analysis. Of these, 113 had baseline proteomic data and day 15 outcomes, and 73 had baseline and day 15 proteomic data as well as day 15 outcomes (Fig. S1).

We used an unsupervised approach to filter proteins in this dataset of 113 patients prior to analysis. We applied a log_2_ transformation to the protein abundances to normalize the data. We then calculated the variances of each log_2_-transformed protein measured at baseline, removing any whose variance fell below the 2.5th percentile (0.0325) and above the 97.5th percentile (1.63), resulting in removal of 366 proteins, leaving 6935 for the analysis. We used a Uniform Manifold Approximation and Projection (UMAP) plot^7^ on the baseline protein values to visualize any clustering.

### Statistical analysis

We searched for candidate proteins and protein pathways that differentiate our primary outcome using resampling, Mann–Whitney U tests, logistic regression with an elastic net penalty, and logistic regression with an overlapping group lasso penalty. Prior to the multivariate analyses, all proteins were standardized to have mean 0 and standard deviation 1 to facilitate interpretation of the results.

### Baseline analysis

We applied Mann–Whitney U tests^8^ to each protein to compare protein expression levels in patients with mild vs. moderate/severe COVID-19. We used a false discovery rate (FDR) correction to adjust for multiple hypothesis testing^9^. Proteins with an FDR-adjusted p-value ≤ 0.05 were considered statistically significant.

A multivariate approach to identify candidate proteins was used to analyze the dataset considering age, sex, BMI, and race as confounders. Resampling techniques were used for statistical rigor and robustness. We split the data into 50 training and testing sets with a 70:30 ratio, stratified according to the primary outcome of disease severity to ensure a similar proportion of patients by severity. For each training set, we fit a multivariable logistic regression model with an elastic net penalty^10^ to assess the conditional effect of each protein on disease severity and to perform variable selection (i.e. identify important biomarkers in predicting the log-odds of moderate/severe COVID-19). We averaged the coefficients from each fit to obtain proteins with consistently non-zero coefficients. For each testing dataset, the test prediction error and the area under the receiver operating characteristic curve (AUC) were obtained to assess model performance. We averaged these metrics across the 50 splits. The penalty parameter was fixed at 0.5 across all splits. We implemented the logistic regression model with elastic net using the *glmnet* package in R^11^.

### Generation of a proteomic signature

To construct our proteomic signature, we identified top low- or moderately-correlated signatures at baseline with potential to predict the primary outcome when combined with clinical covariates. To do this, we combined results from the univariate and multivariate analyses above. Since the elastic net approach could result in strongly correlated proteins, we followed Brzyski et al.^12^ to select low- to moderately-correlated proteins. We began with the top 50 proteins with the largest conditional effect on the primary outcome (i.e. highest absolute coefficients from the elastic net model) given all the other proteins. From this list, we tagged the protein with the smallest p-value from the univariate analyses. Then, proteins that were highly correlated (Pearson’s r > 0.5) with this protein formed a cluster; the tagged protein is a representative of this cluster. We next tagged the protein with the smallest p-value from the univariate analysis that was not included in the first cluster to form the next cluster of proteins that were highly correlated with this protein. We repeated the process of tagging proteins and forming protein clusters until all proteins were in at least one of the clusters. Given our sample size of 113 and to ensure sufficient statistical power to detect differences in patients with mild vs. moderate/severe COVID-19, we only considered a final model with the top 5 signatures and/or clinical covariates.

### External validation of proteomic signature

We validated the prediction estimates from our protein signature model using data from three independent prospective studies: data from Harvard’s Mass General Hospital in Boston, MA, USA (n = 384)^13^, two University of Colorado hospitals, Aurora, CO, USA (n = 99)^14^, and Karolinska University Hospital and Haga Outpatient Clinic, Stockholm, Sweden (n = 63)^15^. In particular, we obtained predicted estimates (i.e. predicted outcomes and log-odds) using the estimated model and the validation datasets. Each protein was standardized to have the same mean and variance in the discovery dataset. Since the dataset from Harvard University categorized the age variable, we created a model using the same 5 protein signatures and clinical covariates as described above while changing the age to the same categorical variable as defined in the dataset from Harvard. For the other two datasets, we kept age as a continuous variable. For prediction estimates, we assessed the AUC from the receiver operating characteristic (ROC) curve, and reported sensitivity, specificity, and F-1 estimates. These estimates were obtained from the optimal cutoff point on the ROC curve (i.e. Youden’s index). We also evaluated the 5-protein signature without clinical covariates.

### Exploratory analysis: protein pathways identification

We conducted two a priori designed exploratory analyses: (1) Identification of protein pathways most associated with the primary outcome and (2) association of protein level change (delta) during the first two weeks of infection and the primary outcome.

To identify protein pathways most associated with the primary outcome, we used an overlapping group lasso penalty^16^ in a logistic regression model. We used the ToppGene Suite^17^ to find pathways to which the proteins in our dataset belonged. Proteins were identified using their Ensembl ID. For each split for our primary analysis, we first fit the overlapping group lasso model on the training set considering 800 possible values for the penalty control parameter. We used fivefold cross validation to obtain the optimal penalty based on cross-validation error. In our primary analysis, we used each split’s optimal penalty and predicted severity on the test set and obtained the corresponding AUC and prediction error. We implemented the logistic regression model with group penalty using the *grpregOverlap* package in R and performed all analyses using R software version 4.0.0 (The R Foundation)^18^.

As an additional exploratory analysis, we were interested in the association of protein level change and moderate/severe disease. The change in proteins during the first two weeks of infection can provide valuable insight into host response to infection and inform an understanding of COVID-19 pathogenesis and potential therapeutic targets. We compared individuals with mild vs. moderate/severe COVID-19 disease using protein expression at baseline and the difference between baseline and day 15 protein expression (termed *delta*). For each protein and in each group, this was calculated as $lo{g}_{2}Day 15 Expression - lo{g}_{2}Baseline Expression$.

## Results

### Proteomic signature development: study population

**Figure 1:**
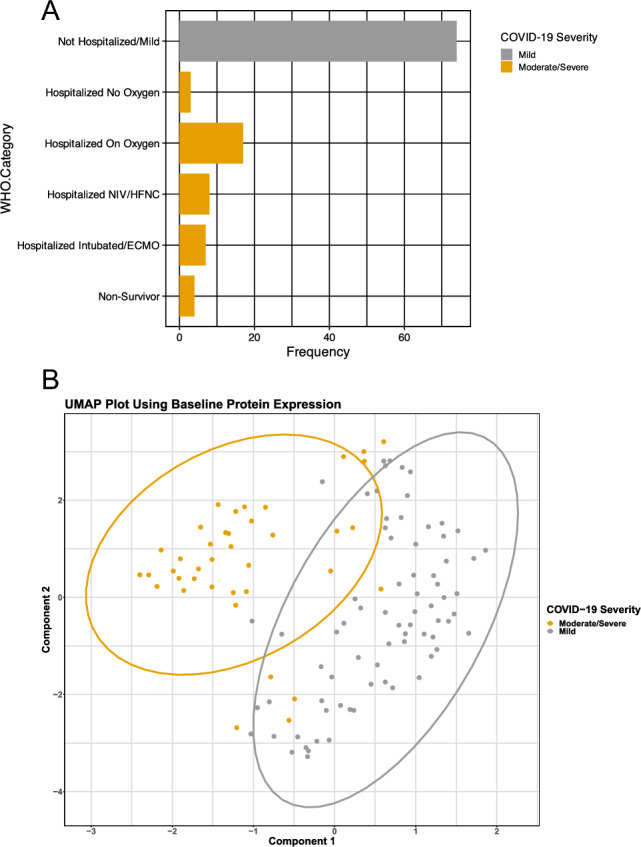
(**A**) Bar graph shows the counts for each WHO modified severity category within the moderate/severe group. (**B**) Uniform manifold approximation and projection plot (UMAP) using baseline protein expression shows clustering of patients with moderate/severe COVID-19 and mild COVID-19.

Table 1 summarizes the demographics of the participants used to generate the proteomic signature. The cohort of patients with moderate/severe COVID-19 (as compared with patients with mild COVID-19) were older in age (median: 58 [IQR: 48–66] vs median: 38.0 [IQR: 26.25–51] and had a higher BMI (median 32 [IQR 26.5–35.1] vs median 26.7 [IQR 24.2–31.6]). A larger percentage of individuals in the moderate/severe group (as compared with patients with mild COVID-19) identified as Black or African American (43.6% [n = 17] vs. 4.1% [n = 3]) or Latinx (17.9% [n = 7] vs 9.5% [n = 7]) and a lower percentage identified as white (28.2% [n = 11] vs. 78.4% [n = 58]). The majority of patients had WHO severity category 5 (not hospitalized) at baseline (Fig. 1A). Figure 1B, summarizes the top 2 components of baseline protein expression highlighting both separation between patients with moderate/severe vs mild COVID-19.

**Table 1:** Demographics of patient sample used in analysis of the association between baseline proteome and COVID-19 disease severity.

	Moderate/severe (N = 39)	Mild (N = 74)	Total (N = 113)	p-value
Sex				
Male	27 (23.9%)	39 (34.5%)	66 (58.4%)	0.09
Age				
Median [IQR]	58.0 [48.0, 66.0]	38.0 [26.25, 51.0]	58.0 [48.0, 66.0]	< 0.001
Race				< 0.001
Asian	1 (2.6%)	3 (4.1%)	4 (3.5%)	
Black or African American	17 (43.6%)	3 (4.1%)	20 (17.7%)	
White	11 (28.2%)	58 (78.4%)	69 (61.1%)	
Latinx	7 (17.9%)	7 (9.5%)	14 (12.4%)	
Other/unknown	3 (7.7%)	3 (4.1%)	6 (5.3%)	
Body mass index (BMI)				0.002
Median [IQR]	32.0 [26.47, 35.10]	26.7 [24.16, 31.58]	28.3 [24.5, 32.84]	
Treatment				0.6
Losartan	21 (18.6%)	36 (31.9%)	57 (50.4%)	
Diabetes				0.015
No	29 (74.4%)	69 (93.2%)	98 (86.7%)	
Yes	9 (23.1%)	5 (6.8%)	14 (12.4%)	
Missing	1 (2.6%)	0 (0%)	1 (0.9%)	
Coronary artery disease				0.3
No	37 (94.9%)	74 (100%)	111 (98.2%)	
Yes	1 (2.6%)	0 (0%)	1 (0.9%)	
Missing	1 (2.6%)	0 (0%)	1 (0.9%)	
Hypertension				< 0.001
No	16 (41.0%)	68 (91.9%)	84 (74.3%)	
Yes	22 (56.4%)	6 (8.1%)	28 (24.8%)	
Missing	1 (2.6%)	0 (0%)	1 (0.9%)	
Atrial fibrillation				0.044
No	34 (87.2%)	73 (98.6%)	107 (94.7%)	
Yes	4 (10.3%)	1 (1.4%)	5 (4.4%)	
Missing	1 (2.6%)	0 (0%)	1 (0.9%)	
Pulmonary hypertension				NA
No	37 (94.9%)	74 (100%)	111 (98.2%)	
Missing	2 (5.1%)	0 (0%)	2 (1.8%)	
Asthma				0.7
No	36 (92.3%)	66 (89.2%)	102 (90.3%)	
Yes	3 (7.7%)	8 (10.8%)	11 (9.7%)	
Chronic bronchitis				NA
No	38 (97.4%)	74 (100%)	112 (99.1%)	
Missing	1 (2.6%)	0 (0%)	1 (0.9%)	
Chronic obstructive pulmonary disease (COPD)				0.012
No	34 (87.2%)	74 (100%)	108 (95.6%)	
Yes	4 (10.3%)	0 (0%)	4 (3.5%)	
Missing	1 (2.6%)	0 (0%)	1 (0.9%)	
HIV				NA
No	38 (97.4%)	74 (100%)	112 (99.1%)	
Missing	1 (2.6%)	0 (0%)	1 (0.9%)	
Uses cigarettes				0.7
No	37 (94.9%)	68 (91.9%)	105 (92.9%)	
Yes	2 (5.1%)	6 (8.1%)	8 (7.1%)	
Use vape products				NA
No	39 (100%)	74 (100%)	113 (100%)

### Identification of proteins with largest conditional effect on moderate/severe COVID-19 infection

#### Univariate analysis

**Figure 2:**
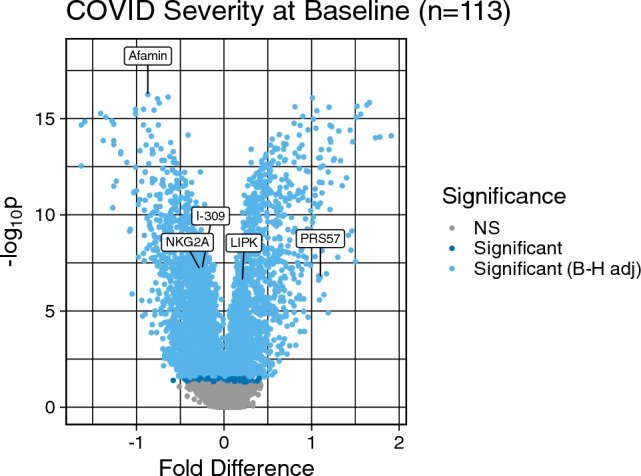
Volcano plot of -log_10_ (p-value) from Mann–Whitney U tests comparing each protein between moderate/severe and mild COVID-19 cases vs. the fold change difference in log_2_ protein expression between moderate/severe and mild COVID-19 cases. Proteins shown in dark blue are significant for COVID-19 severity according to the unadjusted p-value after log_10_ transformation, and proteins shown in light blue are significant according to the adjusted p-value after log_10_ transformation. Proteins selected for the 5-protein signature are labeled. Fold Difference is log2 protein for moderate/severe group—log2 protein for mild group. Values > 0 indicate the protein is elevated in the moderate/severe group. Values < 0 indicate the protein is elevated in the mild group. Values = 0 indicate the same protein levels in the two groups.

At baseline, 4110 out of 6935 proteins were significant at a FDR level of 0.05 comparing patients with mild vs. moderate/severe COVID-19. Figure 2 shows a volcano plot for the negative log_10_ p-value vs. fold change difference for the baseline proteins. 83 proteins are significant after FDR adjustment with a fold change difference magnitude greater than 1, and 17% of proteins (59/83) had higher median expression in moderate/severe cases. We summarize the top 10 baseline proteins in Table S1.

#### Multivariable analysis

Using logistic regression with an elastic net penalty to predict moderate/severe disease, 563 proteins at baseline were selected in at least one of the fifty training/test splits. Table 2 summarizes the top 10 proteins identified as increasing the risk for moderate/severe COVID-19. The average test error rate was 4.7% and the average test AUC was 0.964. The direction of the effects of each protein was consistent across the fifty training/test splits.

**Table 2:** Top 10 selected baseline proteins from logistic regression with an elastic net penalty.

Protein target	Average coefficient	Odds ratio
Coagulation factor IX	0.219	1.245
SMOC1	0.207	1.230
Coagulation factor IXab	0.197	1.218
OAF	0.179	1.196
P-Cadherin	− 0.158	0.854
VWA1	0.142	1.153
RBM18	0.136	1.145
Lymphotoxin a1/b2	− 0.131	0.877
GFRa-1	0.121	1.129
C9	0.120	1.127

Proteins are ordered by the magnitude of their log-odds ratio (coefficient) for moderate/severe COVID-19 averaged across 50 training set splits. Proteins with a higher average coefficient in magnitude have the largest conditional effect on moderate/severe disease, given all other proteins.

### Baseline protein signature

Combining the univariate and multivariate results, we identified 5 proteins with sufficient power to differentiate patients with mild vs. moderate/severe COVID-19. These proteins were Afamin (seq.18196.8), I-309 (seq.13687.5), NKG2A, PRS57, and LIPK. The Pearson correlation between pairs of proteins was low to moderate (r < 0.5) (Fig. S2). A logistic regression model of the outcome with these five proteins resulted in an AUC of 0.9548 and an error rate of 3.54%. The variance inflation factor (VIF) for these proteins ranged from 1.09 to 2.19 which indicated that the proteins were not highly linearly related. For the sake of brevity, we shall use the target names to refer to these protein signatures without further specification.

**Figure 3:**
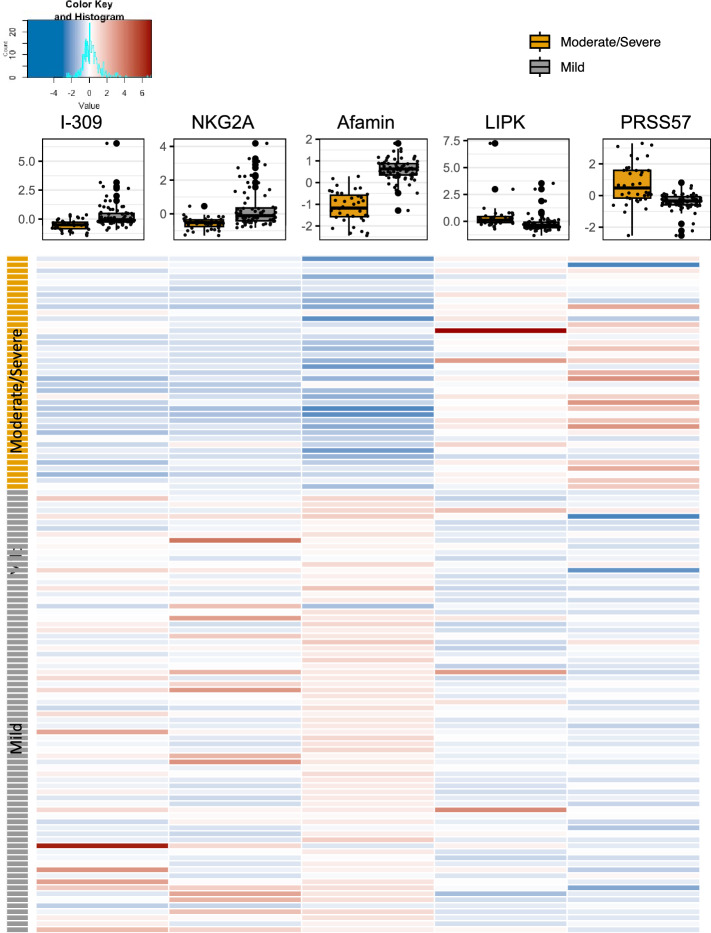
Heatmap and boxplots of the 5-protein signature. Proteins are log2-transformed and scaled. Heatmaps were generated using R Statistical Software v4.2.1.

We further investigated inclusion of the clinical covariates age, gender, BMI, and treatment into this signature and age was ultimately included. BMI and sex were excluded due to convergence issues and high VIFs. Treatment was excluded due to a null effect from the inpatient and outpatient clinical trials. The addition of age to the 5 proteins increased the AUC (0.9804) and reduced the error rate (1.77%). The VIFs were also acceptable (< 5). Figure 3 shows a heatmap of log-transformed, scaled abundances and boxplots of the 5 proteins.

### External validation

**Figure 4:**
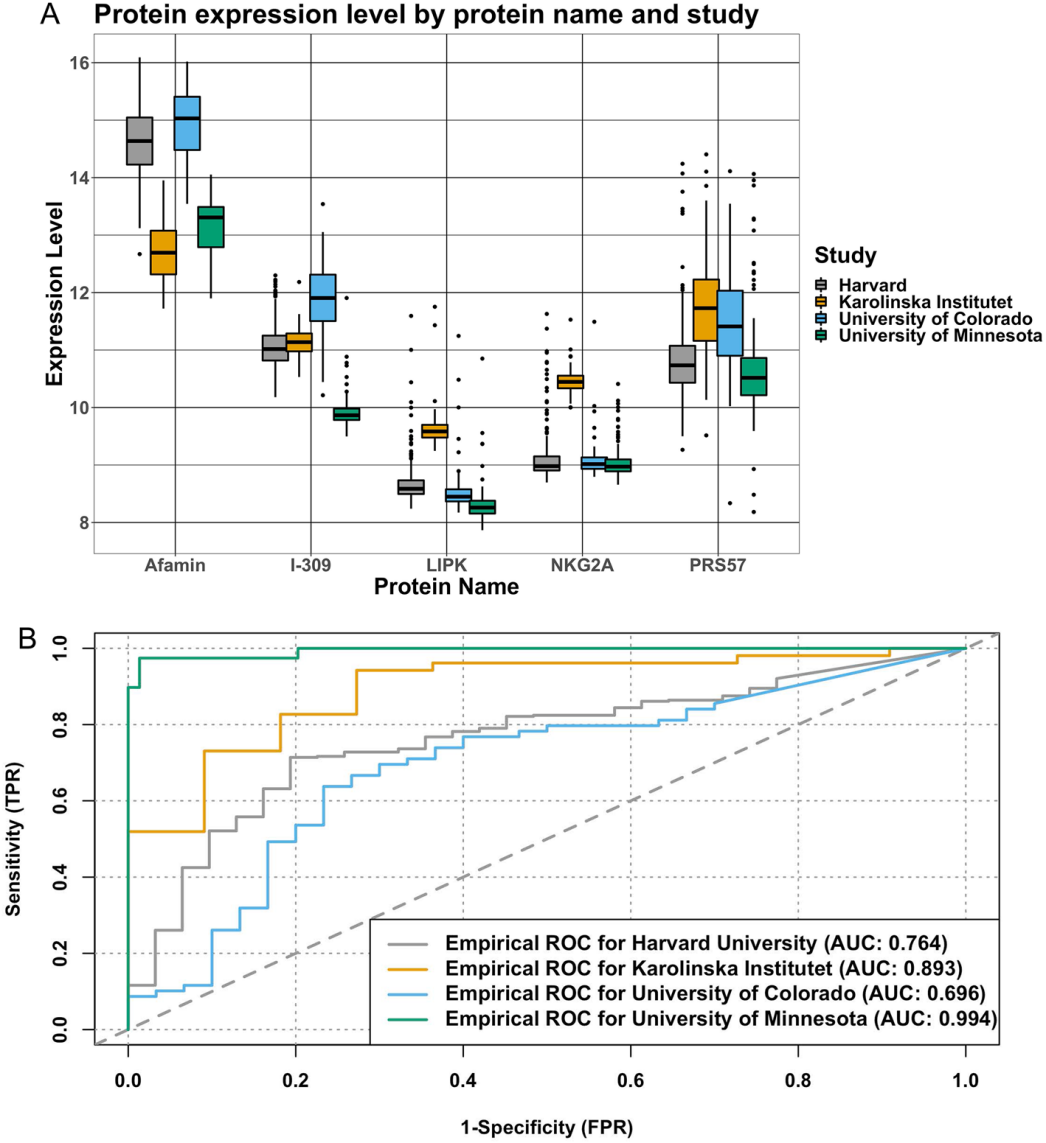
(**A**) Boxplots showing distributions of each protein across the four datasets. (**B**) ROC curves for the model with protein signatures and age using each of the dataset when validating.

Age distribution followed a bell-shaped curve across all 4 studies (Fig. S3a). Figure S3b–h present box-whisker plots for the identified signatures (and the aptamers under the same name) across the studies. Figure 4a shows notable variation in the distribution of each protein across all studies. The AUCs in the three studies are 0.764, 0.696, and 0.893 for the model with the 5 protein signatures and age using datasets from Harvard University, the University of Colorado, and Karolinska Institutet, respectively (Fig. 4b). A model with only the protein signature without any covariates resulted in the following AUCs for the three datasets, respectively: 0.722, 0.715, and 0.876. Sensitivity, specificity, and F-1 score at the cutoff point with optimal Youden index are also shown in Table 3.

**Table 3:** Comparison of AUC, sensitivity, specificity, F1 score, and the cutoff point with optimal Youden index from the model with protein signatures using each dataset as validation.

Dataset	Cases/sample size	AUC	Sensitivity	Specificity	F1 score
**(A)** Model with protein signatures and age					
Minnesota	39/113	0.994	0.974	0.986	0.974
Harvard	353/384	0.764	0.714	0.806	0.825
Colorado	69/99	0.696	0.638	0.767	0.733
Karolinska	52/63	0.893	0.942	0.727	0.942
(B) Model with protein signatures only					
Minnesota	39/113	0.993	0.949	0.987	0.961
Harvard	353/384	0.722	0.589	0.839	0.735
Colorado	69/99	0.715	0.696	0.767	0.774
Karolinska	52/63	0.876	0.865	0.818	0.909

(a) shows the model with both protein signatures and age, and (b) shows the model with protein signatures only.

### Exploratory analysis 1: identification of protein pathways associated with development of moderate/severe COVID-19 infection

From logistic regression with overlapping group lasso penalty models, we identified 54 baseline pathways selected in at least one of the fifty training/test splits. The average test error and average test AUC were 4.4% and 0.9875, respectively. These results were robust to the number of folds considered in obtaining the optimal penalty parameter when we added a ridge penalty in addition to the lasso penalty. The most aberrant pathway identified was dysfunction in the Extrinsic Pathway of Fibrin Clot Formation, specifically coagulation factors VII (lower in patients with moderate/severe disease) and IX (higher in patients with moderate/severe disease) (Fig. S4). In addition to the Extrinsic Pathway of Fibrin Clot Formation, the Scavenging by Class B Receptors pathway was also selected across all 50 data splits (Table 4). Ligand-receptor interactions was selected in 98% of the splits, and PI3K/AKT signaling was selected in 78% of the splits.

**Table 4:** Top selected pathways, ordered by the percentage of 50 training folds selected.

Pathway	Proteins in pathway	% of splits selected
Scavenging by Class B Receptors	APO A-1, APO B, CD 36 ANTIGEN, SCRB1	100
Extrinsic pathway of fibrin clot formation	TF, Coagulation Factor VII, Coagulation Factor IX, Coagulation Factor IXab, Coagulation Factor X, Coagulation Factor Xa, TFPI	100
Ligand-receptor interactions	DHH, CDON, GAS1, IHH, Sonic Hedgehog, BOC, HHIP	98
MET activated PI3K/AKT signaling	GAB1, GRB2 adapter protein, HGF, Met, P85A	78
Melanocyte development and pigmentation pathway	BCL-2, CREB1, SCF sR, SCF, MITF, KS6A1	74
Pyrimidine deoxyribonucleosides degradation	CDA, TP, UPP2, UPP1, SCO2	74
Visceral fat deposits and the metabolic syndrome	Glucocorticoid receptor, DHI1, LPL, PPAR gamma, Retinoic acid receptor RXR-alpha, TNF-a, Resistin, Adinopectin	64
C20 prostanoid biosynthesis	PGD2 synthase, COX-2, THAS, TEBP, PTGD2, PGES2	64
Defective ST3GAL3 causes MCT12 and EIEE15	Aggrecan, Fibromodulin, Lumican, OMD, MIME, KERA	60
Catabolism of glucuronate to xylulose-5-phosphate	CRYL1, DCXR, Sorbitol dehydrogenase, XYLB, AK1A1	56

Proteins in the pathway column lists the proteins available in our dataset that were also part of the selected pathway.

### Exploratory analysis 2: change in protein levels associated with severe COVID-19 infection

#### Univariate analysis

**Figure 5:**
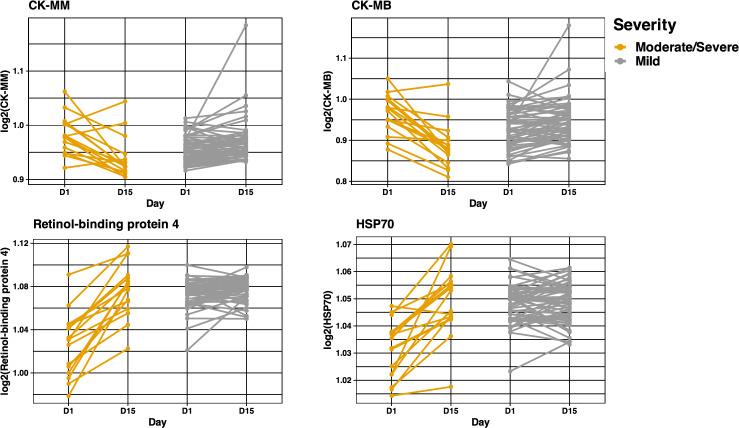
Line plots of the top 4 proteins identified in the delta analysis. *CK-MM* creatine kinase MM, *CK-MB* creatine kinase MB, *RBP-4* retinol binding protein 4. The moderate/severe group has lower levels of CK-MM and CK-MB over 15 days. The moderate/severe group has higher levels of retinol-binding protein 4 and HSP70 over 15 days.

In addition to investigating if baseline protein levels were associated with severity, we also studied if a delta, or change, in protein levels (baseline to day 15) was associated with disease severity by day 15. 1144 proteins were significant after FDR adjustment between baseline and day 15 when comparing mild vs. moderate/severe patients using Mann–Whitney U tests. Figure S5 shows a volcano plot for the negative log_10_ p-values vs. fold change difference for the protein deltas between baseline and day 15. Of note, CK-MB and CK-MM are located at the upper left quadrant, suggesting a highly significant p-value and relatively large log fold change, and lower levels of CK-MB and CK-MM in the moderate/severe group over 15 days. Retinol-binding protein 4 (RBP) and HSP-70 locate to the upper right quadrant, with higher levels observed in the moderate/severe group over 15 days. We summarize the top 10 protein deltas in Table 5 and show plots of these proteins in Fig. 5.

**Table 5:** Top selected protein changes (delta) from logistic regression with an elastic net penalty.

Protein target	Average coefficient	Odds ratio
Afamin	0.178	1.195
Retinol-binding protein 4	0.178	1.194
CK-MB	− 0.156	0.856
sRAGE	− 0.111	0.895
VWA1	− 0.109	0.897
ITI heavy chain H2	0.106	1.112
CK-MM	− 0.102	0.903
Biotinidase	0.101	1.107
HSP 70	0.086	1.090
MYL6B	− 0.0642856	0.938
Endothelin 2	0.0632905	1.065

Proteins are ordered by the magnitude of their log-odds ratio (coefficient) for moderate/severe COVID-19 averaged across 50 training and test set splits. Proteins with a higher magnitude coefficient have the largest conditional effect on moderate/severe disease, given all other proteins.

## Discussion

The objective of this study was to develop and externally validate a baseline prognostic proteomic signature using data from patients that participated in 5 multi-center randomized controlled or prospective biomarker COVID-19 trials. To date, no previously developed prognostic proteomic signatures include data from both outpatients, emergency department, and inpatients with COVID-19. The deliverable of this project, is an internationally validated prognostic signature, which could be used in either the outpatient or inpatient setting. The secondary objective was exploratory, to identify (1) protein pathways and/or (2) protein level changes within the first 2 weeks of acute infection that are associated with the development of moderate/severe (vs mild) disease. During the secondary exploratory analysis we noted the following 3 findings worthy of future investigation: (1) baseline dysregulation of the extrinsic pathway of fibrin clot formation, ligand receptors, PI3K signaling, as well as visceral fat deposits and metabolic pathways, were most often associated with severe disease in patients with COVID-19, (2) a reduction in CK-MB/CK-MM during the initial 2 weeks of COVID-19 infection is associated with severe disease, and an (3) increasing HSP 70 and Retinol-binding protein 4 are associated with severe disease.

Proteomic profiling of sera can serve as a novel predictive biomarker early in the course of infection to predict with high accuracy (AUC 0.98 and error rate of 1.77%) if a patient will develop disease requiring hospitalization. Importantly, this biomarker outperforms published performance metrics of current clinical prognostic models^19–21^. To date, there have been over 13 published proteomic prognostic signatures with AUCs ranging from 0.8 to 0.99^13,22–26^. Similarly, previously described signatures have analyzed plasma (similar to our study) or serum using proximity extension assay (PEA) technology or mass spectrometry-based strategies, whereas in this study we used an aptamer-based proteomic platform. Of the previously published baseline proteomic signatures which sought to predict hospitalization or severe outcomes, only 5 included more than 50 patients. (1) Bauer et al.^24^ enrolled 97 patients that presented to the ED and identified a 5-protein signature ADM, IL-6, MCP-3, TRAIL-R2, and PD-L1 with AUROC 0.8–0.87); however, this was not externally validated. (2) Demichev et al.^27^ enrolled 139 hospitalized patients and developed a proteomic and clinical signature which could detect the need for mechanical ventilation with AUROC 0.99. This signature was then externally validated on a cohort of 99 hospitalized patients (AUROC 0.97). (3) Filbin et al.^13^ enrolled 306 patients that presented to the ED (23 were discharged home, and the rest admitted) and developed a predictive classifier which included the following proteins: IL-6, IL-1RL1, PTX3, IL-1RN, KRT-19, and TRIAP1 which was associated with an AUROC 0.85 to predict severe (mechanical ventilation or death) vs mild/moderate disease. (4) Al-Nesf et al.^23^ enrolled 100 patients that were admitted to the hospital and identified a 12-protein (MSTN, CLEC4C, PTX3, TNC, SMOC1, HGF, IL6, IL1RL1, AREG, KRT19, TNFRSF10B, IL18R1) and 7 clinical test signature which was associated with an AUROC 0.99. This signature was then externally validated using Filbin’s cohort with AUROC 0.84. (5) Finally, Perreau et al.^26^ enrolled 98 patients that were admitted to the hospital and identified a two protein signature (HGF and CXCL13) that predicted severe COVID-19 disease with AUROC 0.9–0.97.

It is important to point out that each of these studies were skewed towards patients that had symptoms severe enough requiring them to present to the ED. Thus it makes sense that the markers they identified which were predictive were also inflammatory cytokines which were likely upregulated at the time the patient presented to the ED and more associated with the development of severe disease and death. However, what is critically needed is a signature that can be used in mildly symptomatic outpatients at baseline and predict which patients will decompensate and require hospitalization. Our study sought to fill this void by leveraging a simultaneously enrolling outpatient biomarker trial in COVID-19. An additional strength of our study is the integration into a randomized controlled trial to ensure homogeneity of patients at study enrollment compared to other studies to date that suffer from heterogeneity in patient comorbidities or treatment received at the time of protein profiling.

### External validation

The AUCs using datasets from the Harvard University and University of Colorado studies are good, although not very high, compared to the AUC in the discovery dataset. Of the external datasets, the prediction of severe disease was lowest in the Colorado dataset (AUROC 0.69). The difference in prediction estimates between these studies might be attributed to the difference in the platforms for proteomic profiling used in the studies. We used the same platform as the Karolinska Institutet study, but our platform was different from the platforms used in the Harvard University and University of Colorado studies. The datasets from the Harvard and University of Colorado studies provide the protein Uniprot IDs but do not provide the Somascan aptamer sequence IDs. As a result, we can match the proteins in our signature using the Uniprot ID but not using the sequence ID so different aptamers may have corresponded to these proteins across studies. As elucidated in Fig. S3b–h, noticeable differences emerge in the distribution of distinct aptamers, which provide a possible explanation of why the performance of our model on the Karolinska dataset is better when compared to the Harvard and University of Colorado datasets. Further, the observed differences in protein levels and age distribution may explain the differences in prediction estimates across the three studies. Figure 4a highlights the distribution of proteins across each dataset. Here we note that the 5 protein levels were all lower in our dataset compared to levels from the Harvard University or the University of Colorado study. One hypothesis for our model's high external validation on the Karolinska data may be due to Afamin, the protein with the highest coefficient estimate in our model. In Fig. 4a, we observe a very similar distribution of Afamin between our dataset and that from Karolinska Institutet. We also noted that Afamin was the only protein from the five signatures that is differentially expressed consistently in all four datasets. Figure S3 highlights the age distribution across datasets. We note that the overall age is higher in the dataset from Karolinska Institutet than the other two datasets. When we categorized age, we observed that the general distribution of ages are similar across the studies. However, the proportions of individuals whose age is between 65 and 79 and whose age is over 80 from the Harvard University study are higher than the other studies. There were no patients with ages below 20 or greater than 80 years old from the Karolinska study, no participants below 20 in the Harvard University study, and no patients over 80 years old in the University of Colorado study.

### Baseline proteomic pathways associated with the development of severe disease

In our study, the most aberrant pathway at baseline that was associated with that development of moderate/severe vs mild disease was dysfunction in the coagulation system, specifically coagulation factors VII (lower in patients with moderate/severe disease) and IX (higher in patients with moderate/severe disease) (Fig. S4). This suggests the specific aberration associated with the development of severe COVID-19 is an overactive intrinsic pathway early in the disease course^28^. We identified patients with moderate/severe disease had significantly reduced Factor VII at baseline, patients with moderate/severe disease also had increased tissue factor pathway inhibitor (TFPI), an inhibitor of the extrinsic pathway (Fig. S4). The intrinsic pathway can be initiated through contact activation (via FXII) or by FXI. Other cohorts^23,29,30^ have found severe illness more strongly associated with abnormal complement and inflammatory protein levels; however, in one of these studies, an elevated plasma level of Factor XII was found to be protective against severe disease^30^, which is congruent with our findings. The ligand-receptor complex pathway, specifically the sonic hedgehog (SHH)-CDON axis, was also frequently dysregulated at baseline between patients who ultimately developed moderate/severe disease and mild disease. In addition to its regulatory roles in embryonic development, the hedgehog family proteins continue to direct homeostasis and turnover of certain cell types in adults^31–34^, and dysregulation of SHH signaling has previously been implicated as a potential pathway driving loss of smell in COVID-19^34,35^. Our findings support other studies which have also identified aberrant PI3K/AKT signaling as one of the most associated pathways in patients that develop severe COVID-19^36,37^. The PI3K/AKT pathway is a potent activator of mTOR and NF-κB signals as well as platelet activity, and activation of PI3K may be effected via angiotensin II signaling^38,39^. Biophysical modeling has predicted that mTOR inhibition disrupts the SARS-CoV-2 viral life cycle^40,41^. Finally, we observed significant dysregulation in visceral fat deposit and metabolic pathways, a finding may provide biologic plausibility for the elevated risk of severe disease course associated with obesity and male sex as both are associated with increased visceral fat stores^42^.

### Change in protein level associated with the development of severe disease

One of the a priori objectives of this study was to characterize the proteome change (or delta) from enrollment through day 15. Identifying temporal trends in biomarkers which are most associated with severe disease allows development of prognostic tools and provides targets to guide future therapeutics. Previous studies have demonstrated an association between elevations of multiple isoforms of CK-MB at initial evaluation and increased odds of severe COVID-19 disease^43–48^. However, whereas there is an overlap between entry CK-MB and CK-MM for mild and moderate/severe disease groups, the reduction in these biomarkers over 15 days was most strongly associated with moderate/severe disease. The overlap with the muscle pathology, post-ICU syndrome and ICU-acquired weakness seem to be vast^49,50^, and while mechanistic understanding the preponderance of autopsy studies point to an immune mediated myopathy which may be related to the interferon I response rather than hypoxic injury. As the degree of CK elevation is likely related to both a patient’s overall muscle mass and the state of their kidney function, the change in CK over time, rather than a binary ‘elevated’ or ‘normal,’ appears to provide better differentiation of disease outcomes. This is of great interest as CK isoforms is a common and readily available assay.

### Limitations

This study is subject to the following limitations. First, patients for the proteomic signature development were included from two different randomized controlled trials. As each trial was run by the same team, the inclusion/exclusion criteria were nearly identical; the only difference was that one trial enrolled symptomatic outpatients with COVID-19. Second, this was a drug trial where patients were randomized to receive losartan vs. placebo; thus, it is possible when doing the delta analysis that patients were influenced by the study drug. However, it is important to note that in one of the trials that investigated pharmacokinetic and pharmacodynamic effects of losartan, losartan did not have significant downstream effects on angiotensin 2, angiotensin-[1-7], ACE or ACE2 within the first 15 days of treatment. Despite this, it is possible given losartan’s inhibition on the angiotensin 1 receptor that there were other pharmacodynamic effects not investigated in the initial trial. Third, the analysis took an agnostic approach with a well-curated but not selective patient cohort. We were interested in patients’ baseline proteomic profiles using samples collected at the time of their first healthcare encounter. We recognize there is variation from patient to patient when COVID-19 presentation arises and when a patient might seek out healthcare. Fourth, applying our signature in other sample cohorts provided insight into its performance, though evaluating these proteins with other assays is needed to address platform bias. Validation of the 5-protein signature and other key results with an orthogonal platform is a necessary future direction. Finally, our analysis did not consider samples from those without COVID-19 so it is unclear if our findings are exclusive to COVID-19 infection, and if not, to what extent our findings differ between those with and without COVID-19. A future analysis investigating the ability of this signature to discriminate between individuals with and without COVID-19 would be worthwhile.

## Conclusion

In this study we developed and externally validated a baseline COVID-19 proteomic signature associated with disease severity for potential use in both outpatients and inpatients with COVID-19.

### Supplementary Information

Supplementary Information.

## Data Availability

Clinical trial data is available from the corresponding author on reasonable request and also publicly available on Vivli-Registry ID NCT04312009 (https://vivli.org/resources/resources/).
