# Author Correction: Development of a proteomic signature associated with severe disease for patients with COVID-19 using data from 5 multicenter, randomized, controlled, and prospective studies

**DOI:** 10.1038/s41598-024-67607-4

**Published:** 2024-07-16

**Authors:** Sandra Castro-Pearson, Sarah Samorodnitsky, Kaifeng Yang, Sahar Lotfi-Emran, Nicholas E. Ingraham, Carolyn Bramante, Emma K. Jones, Sarah Greising, Meng Yu, Brian T. Steffen, Julia Svensson, Eric Åhlberg, Björn Österberg, David Wacker, Weihua Guan, Michael Puskarich, Anna Smed-Sörensen, Elizabeth Lusczek, Sandra E. Safo, Christopher J. Tignanelli

**Affiliations:** 1 Division of Biostatistics, School of Public Health, University of Minnesota, Minneapolis, MN USA; 2 Department of Medicine, University of Minnesota, Minneapolis, MN USA; 3 Department of Surgery, University of Minnesota, 420 Delaware St SE, Minneapolis, MN 55455 USA; 4 School of Kinesiology, University of Minnesota, Minneapolis, MN USA; 5 Division of Immunology and Allergy, Department of Medicine Solna, Center for Molecular Medicine, Karolinska Institutet and Karolinska University Hospital, Stockholm, Sweden; 6 Department of Emergency Medicine, University of Minnesota, Minneapolis, MN USA; 7 Department of Emergency Medicine, Hennepin County Medical Center, Minneapolis, MN USA; 8 Institute for Health Informatics, University of Minnesota, Minneapolis, MN USA

Correction to: *Scientific Reports* 10.1038/s41598-023-46343-1, published online 20 November 2023

The original version of this Article contained an error in the spelling of the author Brian T. Steffen which was incorrectly given as Brian Steffen.

The original Article has been corrected.

