# Supplementary material for: Development of a proteomic signature associated with severe disease for patients with COVID-19 using data from 5 multicenter, randomized, controlled, and prospective studies
Source: Sci Rep. 2023 Nov 20;13:20315. doi: 10.1038/s41598-023-46343-1 (PMC10661735; doi:10.1038/s41598-023-46343-1)
Supplement: Supplementary file 1 — Supplementary Information. [file 41598_2023_46343_MOESM1_ESM.pdf]

Supplemental Files

eFigure 1. Data processing flow chart prior to statistical analysis.

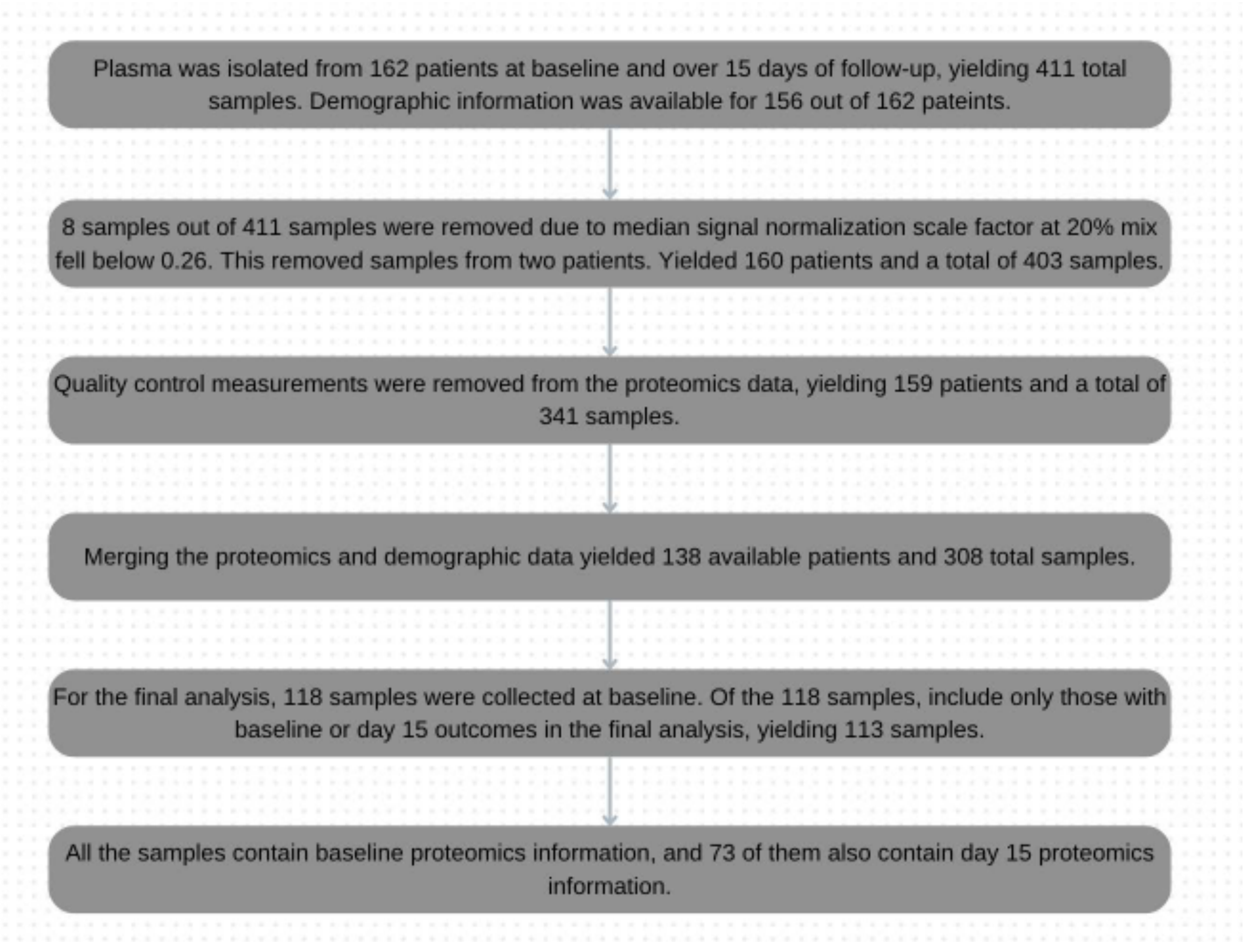

eFigure 2: Correlation plot for proteins included in 5-protein signature shows proteins were not highly correlated. Afamin, I-309, NKG2A, PRS57, and LIPK serve as a 5-protein signature (including age) that predicts mild vs. moderate/severe COVID-19 disease. These proteins were chosen to serve as a signature because they are low- or moderately-correlated with each other and predict disease severity. The correlation is calculated using Pearson correlation.

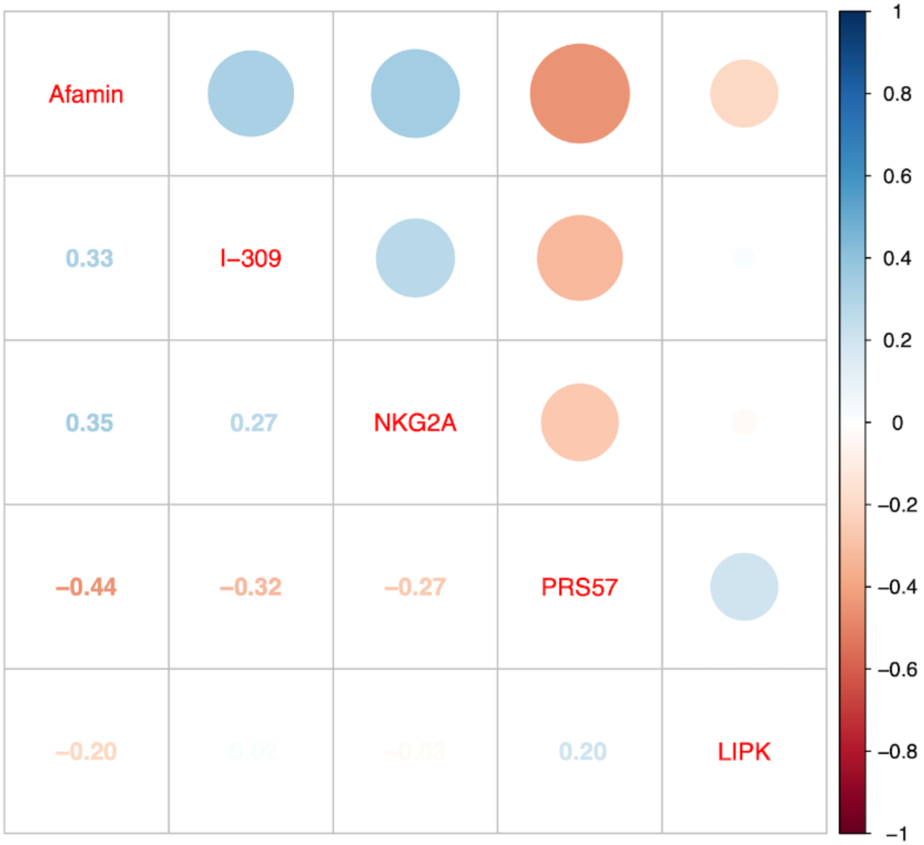

eFigure 3A: Proportions of each age group in each of the four datasets. Barplot showing distributions of age across the four datasets. The groups 0-5 represent age (0) below 20, (1) 20-34, (2) 36-49, (3) 50-64, (4) 65-79, (5) over 80.

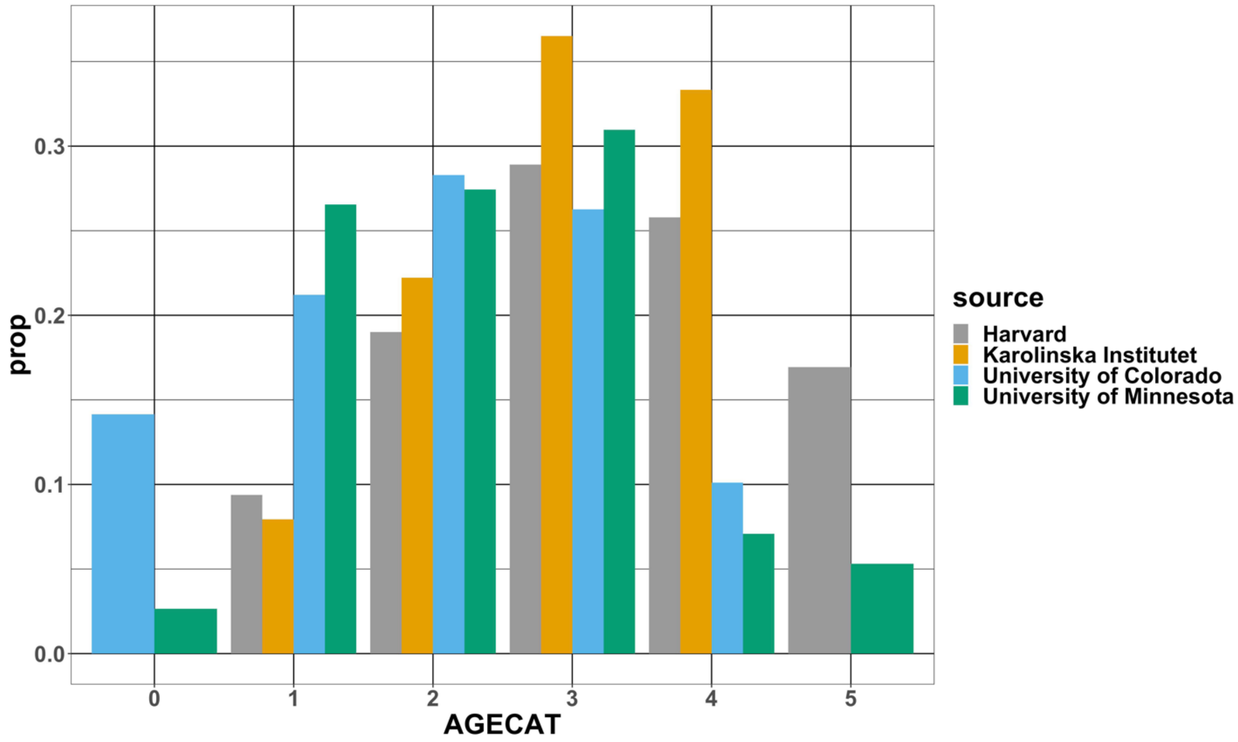

eFigure 3B-H: Box-whisker plots for the identified signatures (and the aptamers under the same name) across the studies. showing distribution of **(B) Afamin (seq.18196.8), (C) Afamin (seq.4763.31), (D) I-309 (seq.13687.5), (E) I-309 (seq.2770.51), (F) NKG2A, (G) LIPK, and (H) PRS57**, respectively, by datasets and Severity.

Since sequence IDs are not provided for the Harvard and the University of Colorado studies, we include plots of seq 4763.31 (afamin, 3C) and seq 2770.51 (I309, 3E). The variables identified in our 5-protein signature were seq.18196.8 (afamin) and seq.13687.5 (I309)

3B

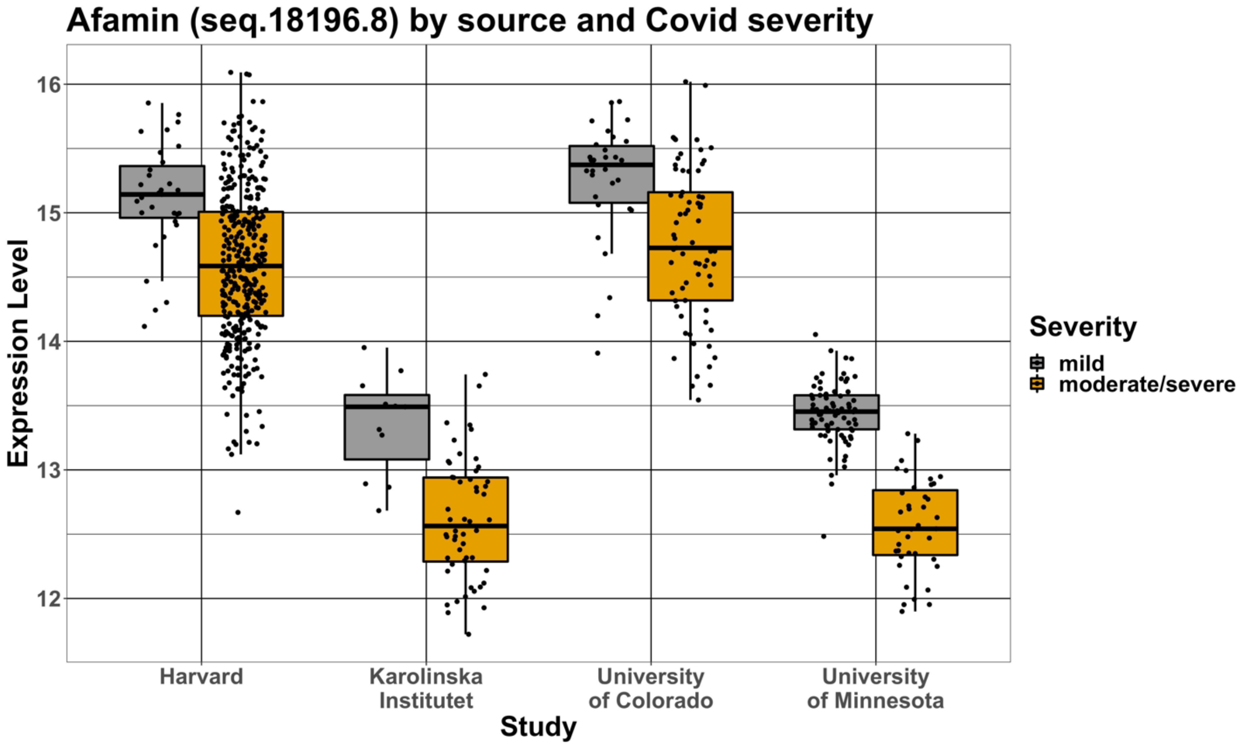

3C

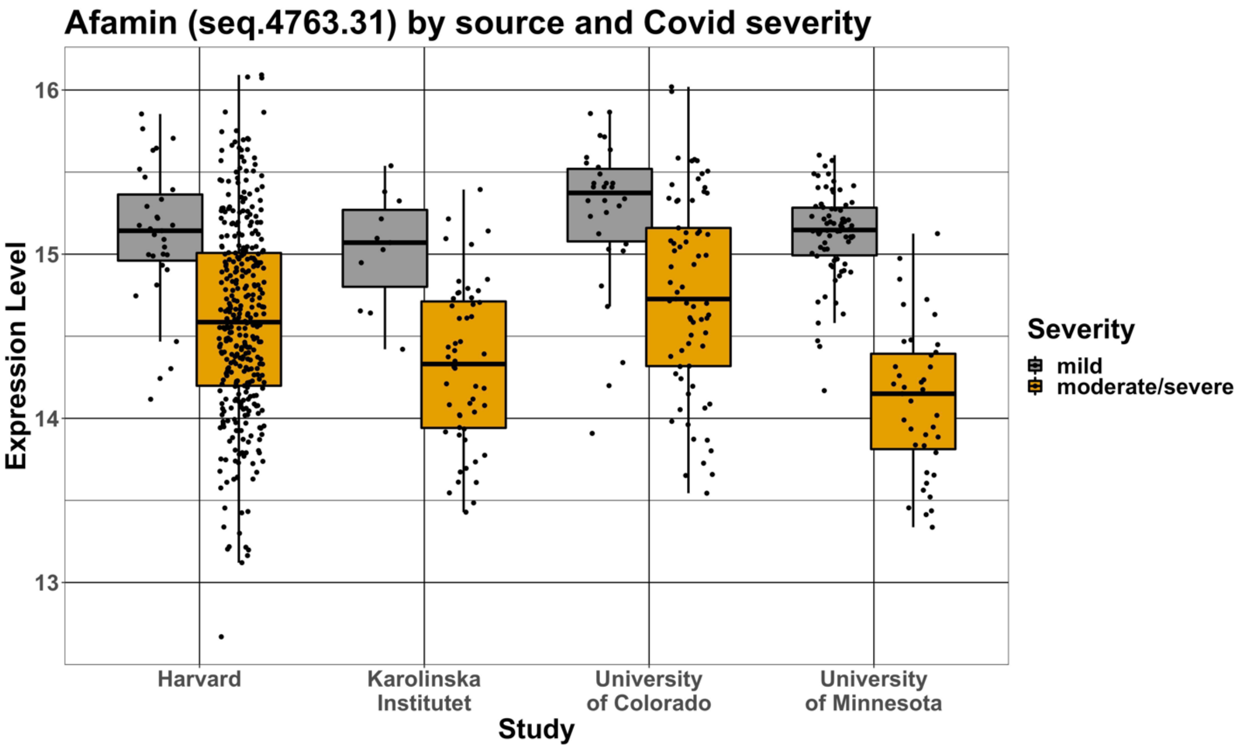
Afamin (seq.4763.31) by source and Covid severity
16
15
14
13
Expression Level
Harvard
Karolinska Institutet
University of Colorado
University of Minnesota
Study
Severity
mild
moderate/severe

3D

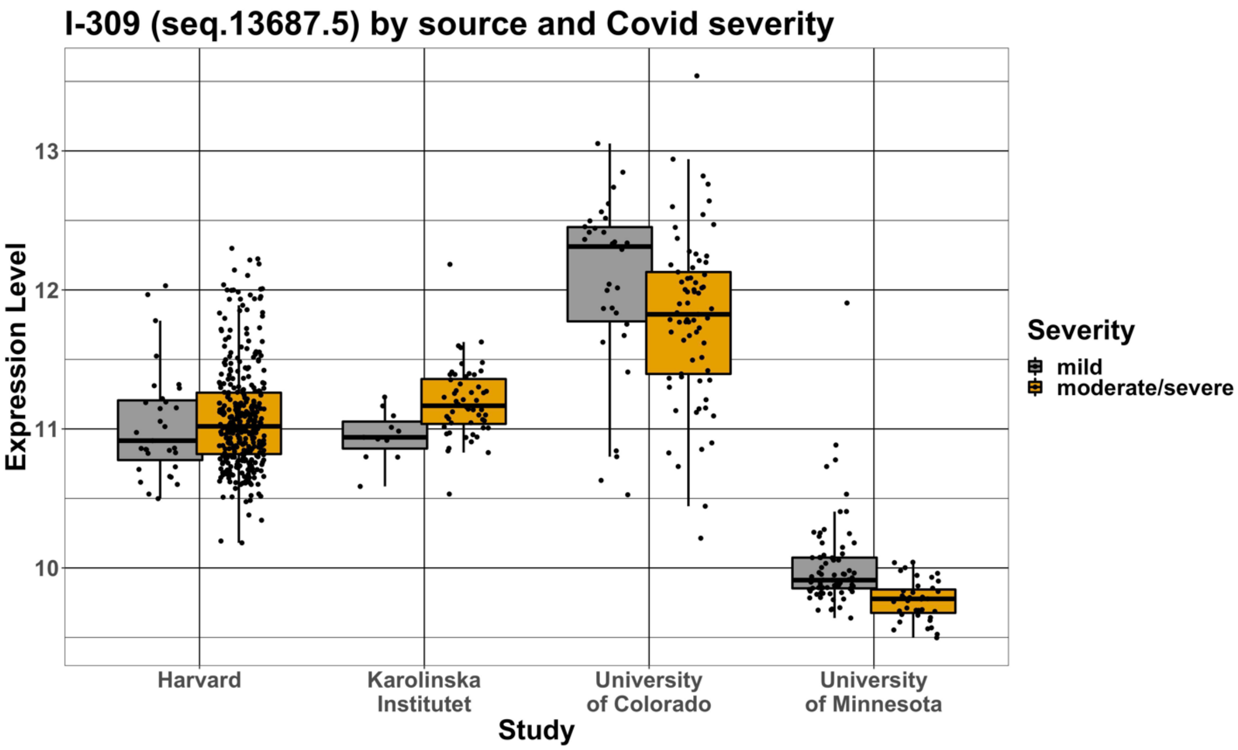
I-309 (seq.13687.5) by source and Covid severity
Expression Level
13
12
11
10
Harvard
Karolinska Institutet
University of Colorado
University of Minnesota
Study
Severity
mild
moderate/severe

3E

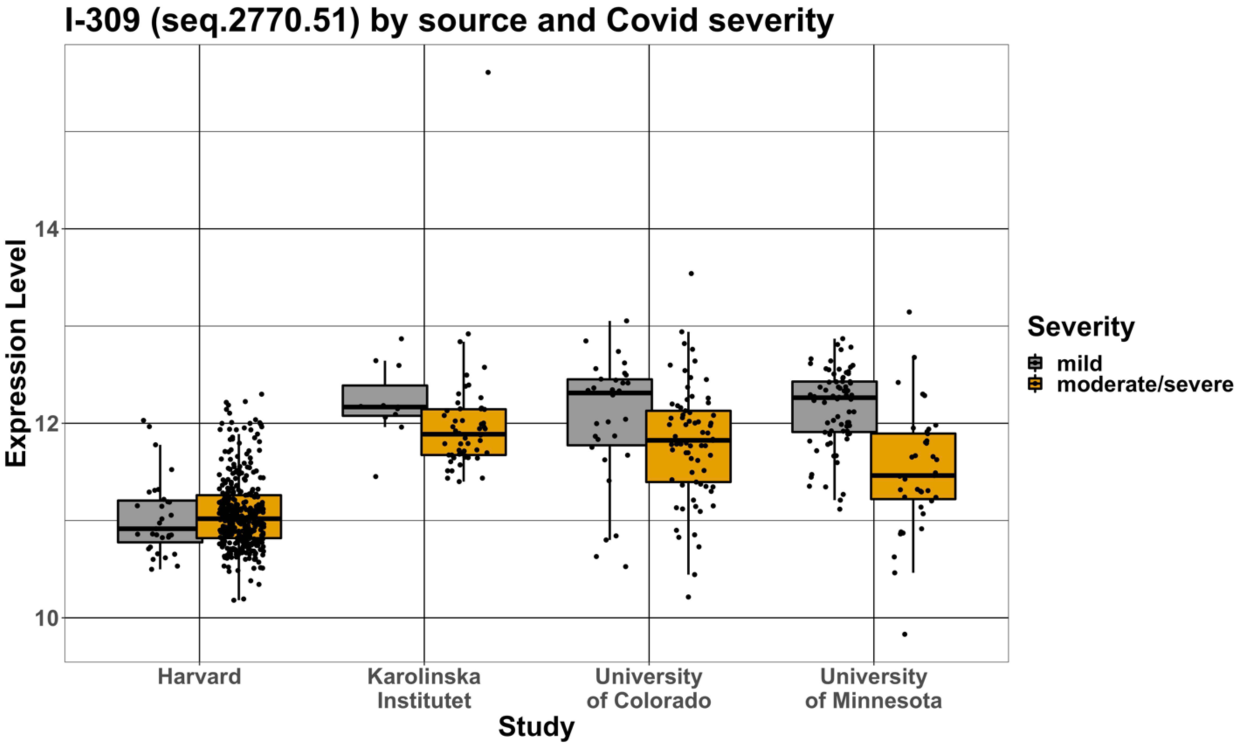
I-309 (seq.2770.51) by source and Covid severity
Expression Level
14
12
10
Harvard
Karolinska Institutet
University of Colorado
University of Minnesota
Study
Severity
mild
moderate/severe

3F

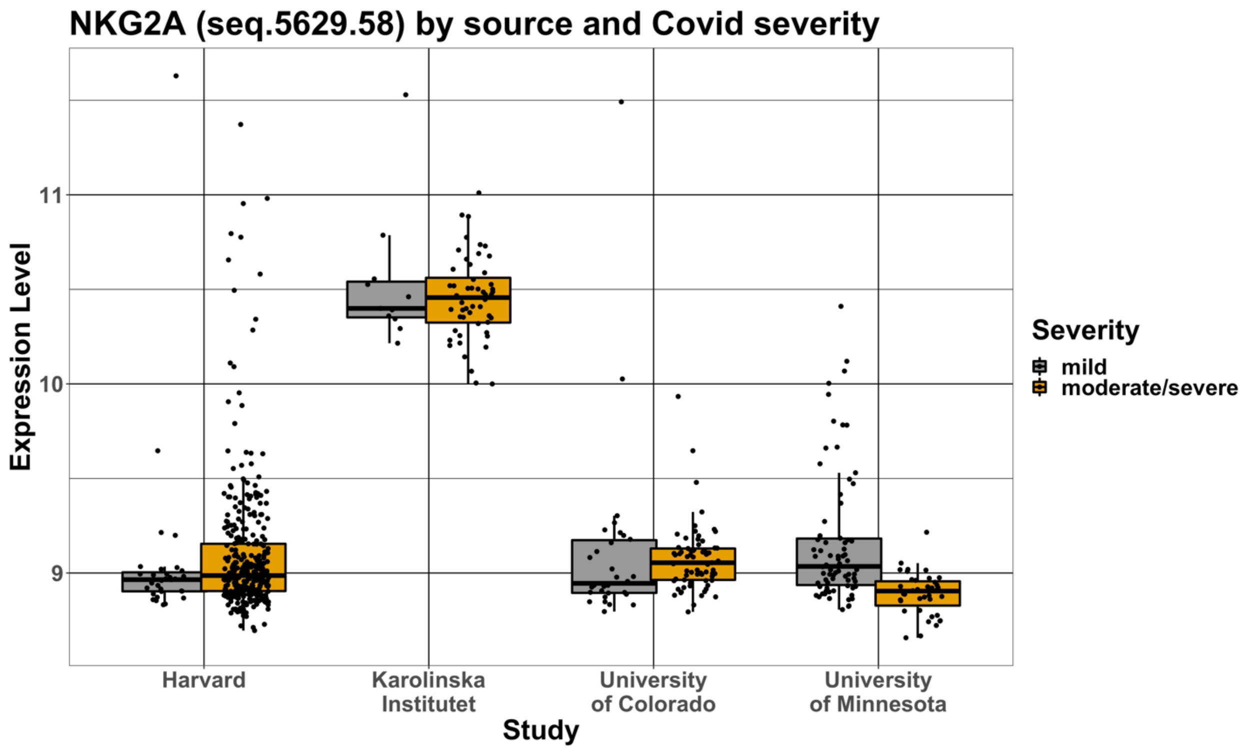
NKG2A (seq.5629.58) by source and Covid severity
Expression Level
11
10
9
Harvard
Karolinska Institutet
University of Colorado
University of Minnesota
Study
Severity
mild
moderate/severe

3G

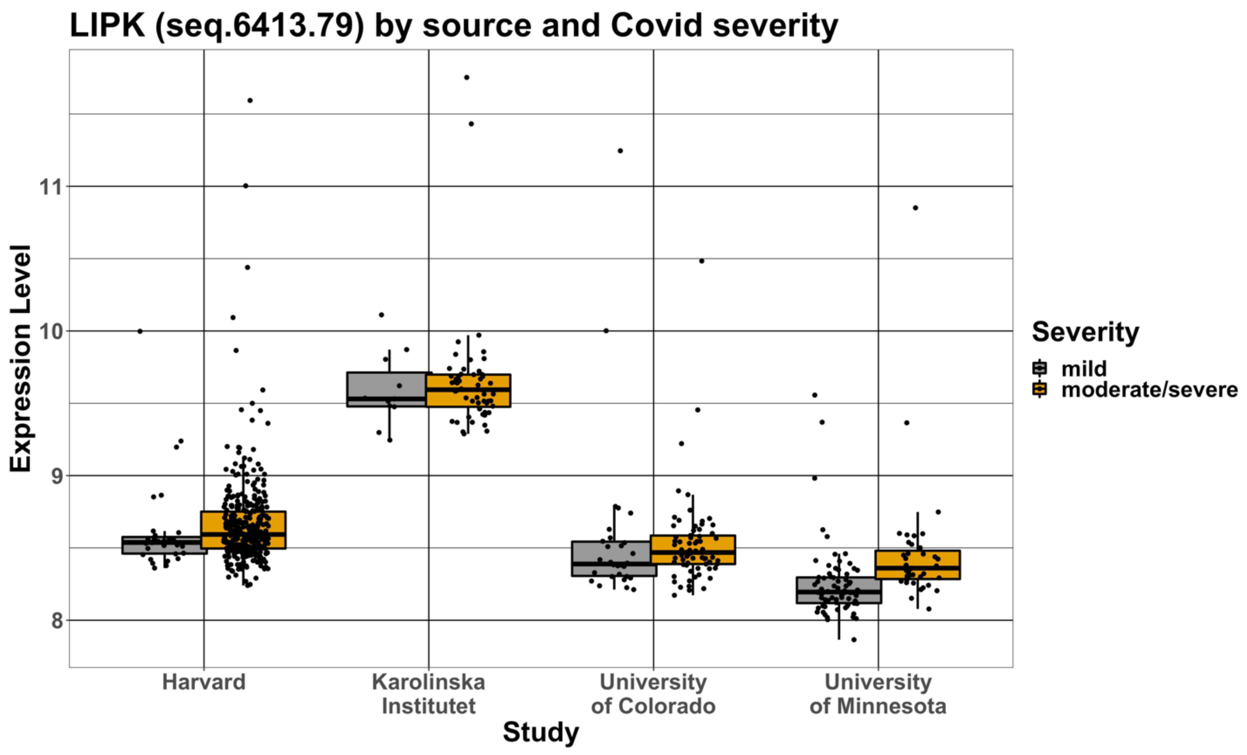
LIPK (seq.6413.79) by source and Covid severity
Expression Level
11
10
9
8
Harvard
Karolinska Institutet
University of Colorado
University of Minnesota
Study
Severity
mild
moderate/severe

3H

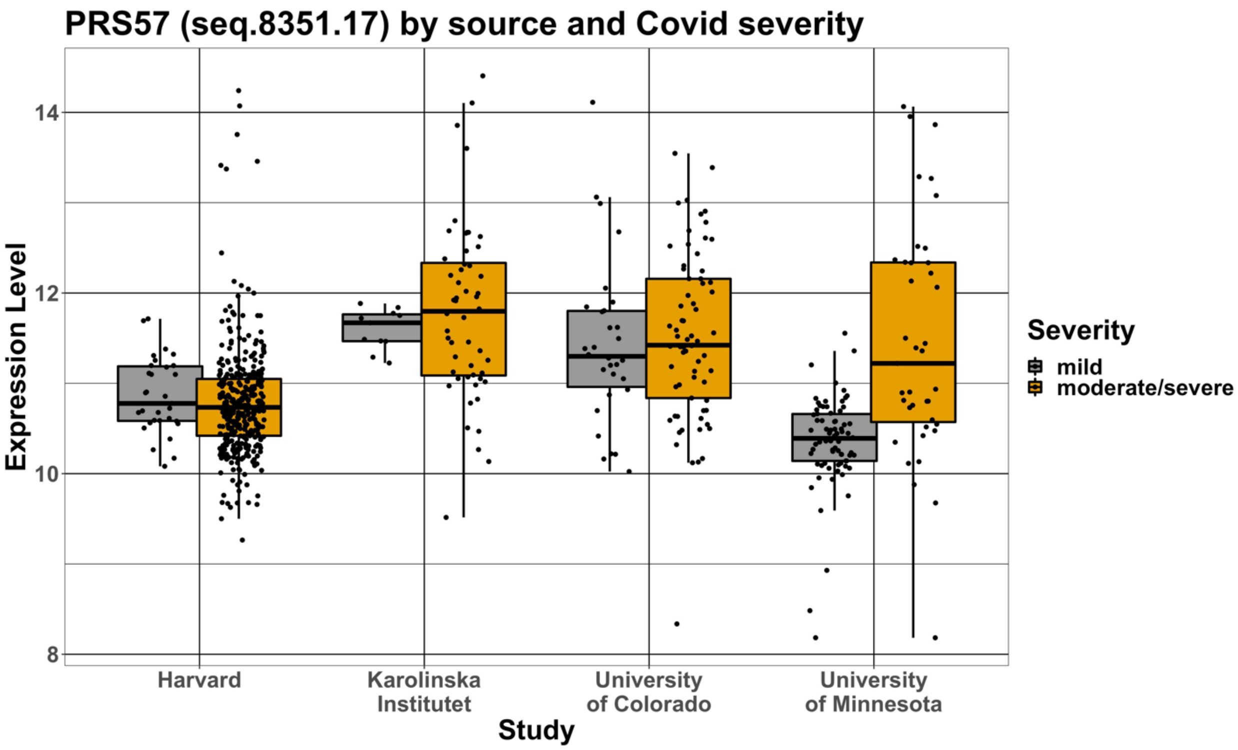

eFigure 4: Protein expression at baseline for proteins in the extrinsic pathway of fibrin clot formation. Violin plot shows the distribution of expression of each protein in the extrinsic pathway of fibrin clot formation for mild vs. moderate/severe COVID-19 patients. Coagulation factors VII, IX, IXab, and TFPI were all significantly different after adjusting for age, sex, BMI, and treatment.

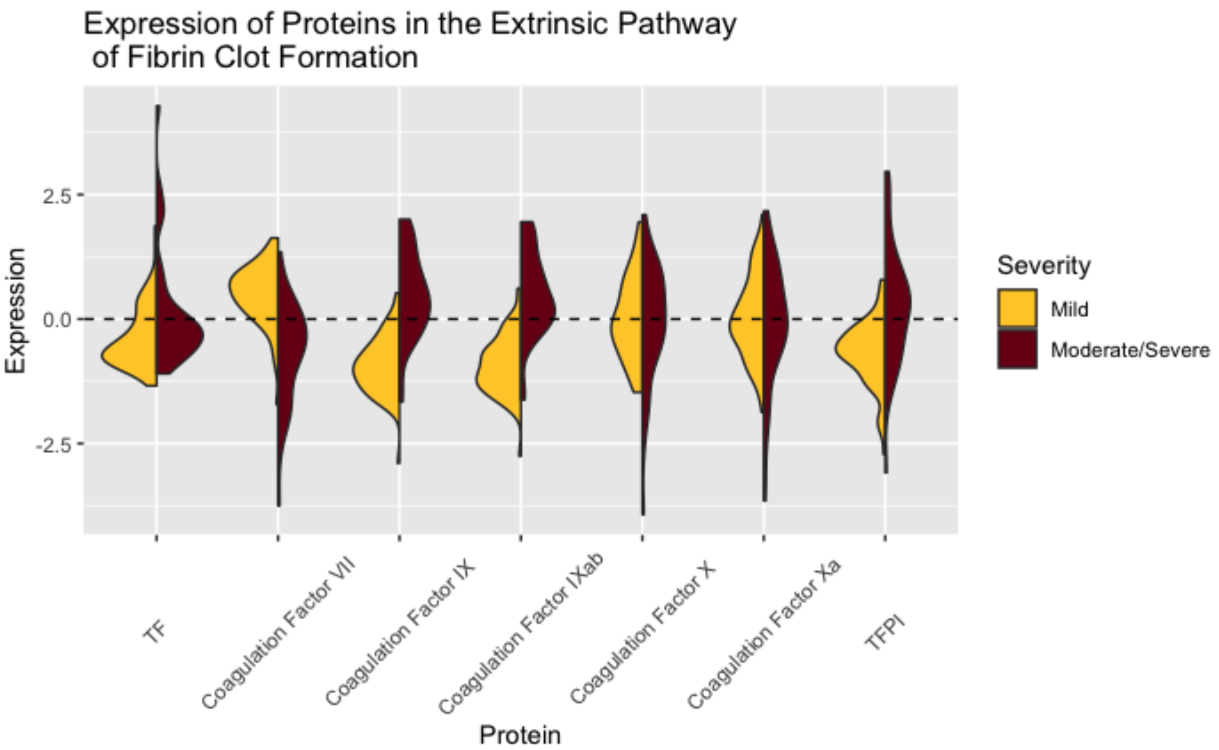

eFigure 5. Volcano plot of Mann-Whitney U test p-values from comparisons of protein deltas in patients with mild vs. moderate/severe COVID-19 disease. Proteins shown in dark blue are significant for COVID-19 severity according to the unadjusted p-value after log10 transformation, and proteins shown in light blue are significant according to the adjusted p-value after log10 transformation. Fold difference is calculated as: (log2 day 15 protein - log2 baseline protein)_moderate/severe - (log2 day 15 protein - log2 baseline protein)_mild. Values >0

indicate the protein is elevated in the moderate/severe group over 15 days. Values< 0 indicate the protein is elevated in the mild group over 15 days. Values=0 indicate the same protein levels in the two groups over 15 days. CK-MM = creatine kinase MM, CK-MB = creatine kinase MB, RBP-4 = retinol binding protein 4

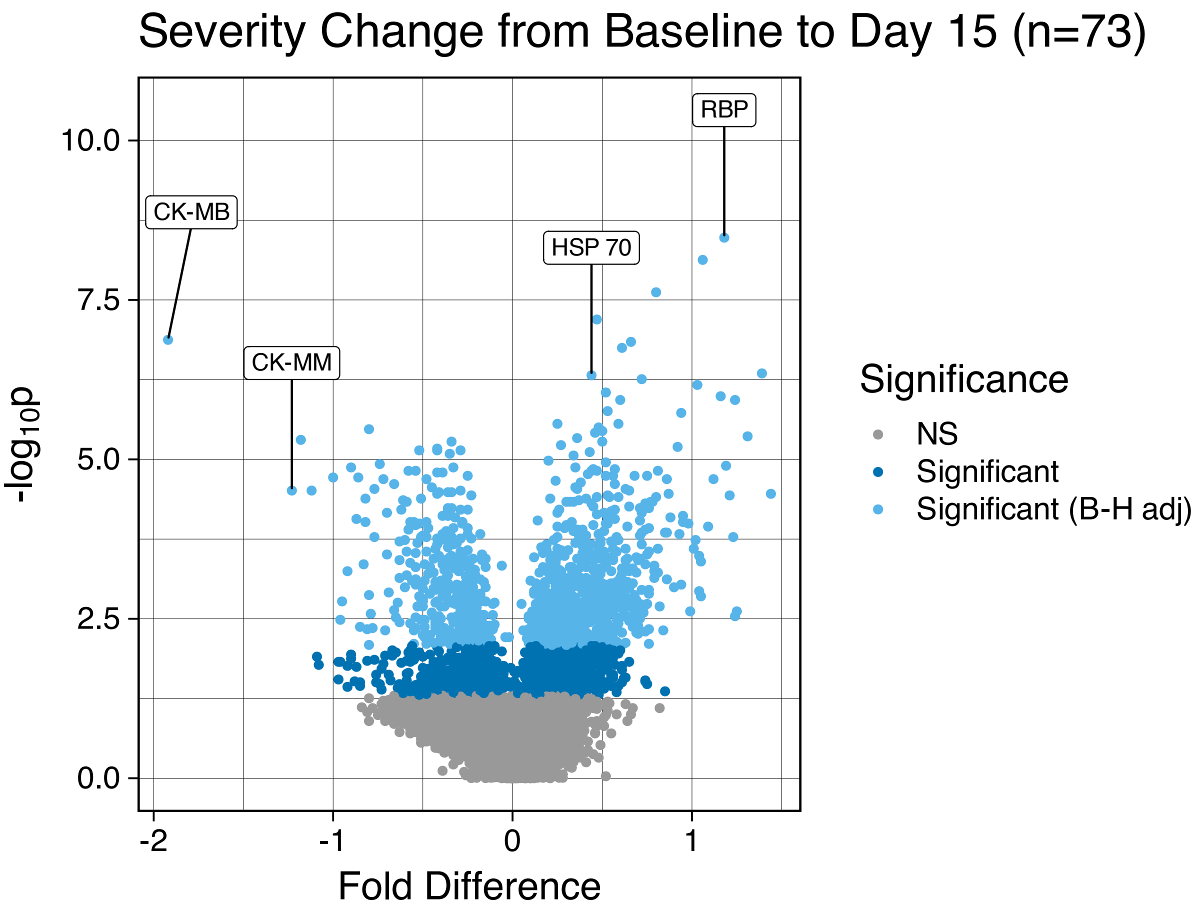

eTable 1: Top 10 proteins most significantly different proteins between moderate/severe and mild COVID-19 cases. Proteins are ordered by p-value.

Protein Target	Fold Change Difference	P-Value	Adjusted P-Value
Afamin	-0.87	5.59e-17	1.62e-13
SET	-0.64	7.60e-17	1.62e-13
ITIH3	1.01	8.42e-17	1.62e-13

a2-HS-Glycoprotein	-0.76	9.32e-17	1.62e-13
SMOC1	1.66	1.47e-16	1.78e-13
Tetranectin	-0.74	1.55e-16	1.78e-13
p130	1.63	1.99e-16	1.78e-13
VSIG4	1.52	2.32e-16	1.78e-13
RBM9	0.81	2.43e-16	1.78e-13
alpha-1-antichymotrypsin complex	1.20	2.56e-16	1.78e-13
